# Probabilistic RNA designability via interpretable ensemble approximation and dynamic decomposition

**DOI:** 10.1093/bioinformatics/btag246

**Published:** 2026-07-07

**Authors:** Tianshuo Zhou, David H Mathews, Liang Huang

**Affiliations:** School of EECS, Oregon State University, Corvallis, OR 97330, USA; Department of Biochemistry & Biophysics, University of Rochester Medical Center, Rochester, NY 14642, USA; Center for RNA Biology, University of Rochester Medical Center, Rochester, NY 14642, USA; Department of Biostatistics and Computational Biology, University of Rochester Medical Center, Rochester, NY 14642, USA; School of EECS, Oregon State University, Corvallis, OR 97330, USA; Department of Biochemistry & Biophysics, Oregon State University, Corvallis, OR 97330, USA

## Abstract

**Motivation:**

RNA design, also known as RNA inverse folding, aims to find RNA sequences that fold into a target secondary structure. However, recent work has shown that some target structures are provably *undesignable*, where no RNA sequence can fold into it as the *minimum free energy* (MFE) structure. In this paper, we go beyond this binary, MFE-based designability and explore a soft, probability-based designability that upperbounds the Boltzmann probability of any design and quantifies how easily or likely any design might possibly fold into the target structure. We introduce a theory of ensemble approximation and a probability decomposition framework for bounding the folding probabilities of RNA structures and motifs in an explainable way. We further develop a linear-time dynamic programming algorithm that efficiently searches over exponentially many decompositions. Combining ensemble approximation with dynamic decomposition search, our method efficiently identifies the optimal motif decomposition that yields the tightest probabilistic bound for a given structure. Our framework is applicable to any *factorizable* energy model or scoring function that decomposes onto loops.

**Results:**

Applying our work, LinearDecompose, to both native and artificial RNA structures in the ArchiveII and Eterna100 datasets, we obtained much tighter probability bounds than baselines. Our work also provides anatomical tools for analyzing RNA structures and pinpointing the sources of design difficulty at the motif level.

**Availability and implementation:**

Source code and data are available at https://github.com/shanry/RNA-Undesign.

## 1 Introduction

RNA secondary structures play crucial roles in the functions of non-coding RNAs such as rRNA (Doudna and Cech 2002) and tRNA (Coller and Ignatova 2024). RNA design, also known as RNA inverse folding, aims to find one or more RNA sequences that fold into a given target structure, typically using the default Turner energy model (Mathews and Turner 2006, Turner and Mathews 2010). However, not all RNA structures are designable (Aguirre-Hernández *et al.* 2007, Yao 2021, Zhou *et al.* 2024, 2025, 2026, Malik *et al.* 2026). Our recent works RIGEND (Zhou *et al.* 2024) and FastMotif (Zhou *et al.* 2025) proved that many native structures in the ArchiveII dataset and many artificial structures in the Eterna100 benchmark are undesignable, in the sense that no sequence can ever fold into them as MFE structures. However, these methods only study the MFE-based criteria and therefore only provide a *binary* notion of designability. As a result, they cannot address the more important ensemble-level design objectives such as equilibrium probability which, by considering competing structures, is more appropriate for RNA design (Zadeh *et al.* 2011, Ward *et al.* 2023, Zhou *et al.* 2023, Tang *et al.* 2026).

This perspective motivates a shift from binary MFE-based criteria to a probabilistic characterization of designability. While MFE-based undesignability can certify that a structure is impossible to be a minimum free energy structure of any RNA sequence, it cannot quantify *how likely* or how easily a sequence might possibly fold into that structure. For example, consider two structures $y_{1}$ and $y_{2}$ that are both undesignable under the MFE criterion. It is nevertheless possible that there exists a sequence $x_{1}$ where $p(y_{1}|x_{1})=0.2$, whereas no sequence can make $p(y_{2}|x)$ exceed 0.01. This discrepancy is invisible to MFE-based analysis but is crucial for understanding practical designability of a target structure. These observations motivate the study of *probabilistic designability*, defined as an upper bound on $p(y^{⋆}|x)$ over all sequences x for a given target structure $y^{⋆}$. The tighter the upper bound, the better we understand the designability of that structure.

Despite its importance, probabilistic designability has received little attention, with CountingDesign (Yao *et al.* 2019, Yao 2021) being the only existing method that addresses this question. However, it relies on exhaustive enumeration: for a given RNA structure or motif, it enumerates and folds all (partial) sequences that satisfy its base-pairing constraints and record the maximum equilibrium probability observed. This brute-force strategy is neither scalable nor interpretable, requiring weeks of computation for structures or motifs of length up to 14.

To bridge the gap between MFE-based and probabilistic designabilities, we propose two main ideas: *ensemble approximation* and *probability decomposition*. Ensemble approximation represents the full RNA folding ensemble using a small, explicitly identified set of rival structures (motifs) that compete with the target structure. This approximated ensemble enables us to derive rigorous upper bounds on folding probability while maintaining full explainability, as each bound is supported by concrete rival structures that thermodynamically dominate the target. Furthermore, we prove that the probability bound of a target structure is no greater than the *product of the local probability bounds* of its constituent motifs under *any* structural decomposition. In order to explore as many decompositions as possible, we develop a *linear-time* dynamic programming algorithm that explores *exponentially many* decompositions to find the one with the tightest upper bound. Our contributions are:

1. **Theory.** We propose a novel and elegant theory of ensemble approximation to interpretably characterize probabilistic RNA designability. We also prove that the probability bound of a structure can be factored into products of local bounds of structural motifs, revealing a more nuanced connection between local and global designabilities.
2. **Algorithms.** We develop efficient algorithms (Algorithms 1 and 2) for approximating Boltzmann ensembles using rival structures or motifs, and introduce a linear-time dynamic programming approach (Algorithm 3) that efficiently explores exponentially many decompositions to obtain the tightest probability bound.
3. **Application.** Applying our methods to both native and artificial RNA structures in the ArchiveII and Eterna100 datasets, we obtained probability bounds that are much tighter than prior approaches. In addition, our methods further provide anatomical tools for analyzing RNA structures and understanding the sources of design difficulty at the motif level.

## 2 Preliminaries: RNA structures and motifs

An RNA sequence x of length *n* is a string of nucleotides $x_{1}x_{2}\ldots x_{n}$, where $x_{i}\in \{A,\;C,\;G,\;U\}$. A secondary structure P for x is a set of paired indices where each pair (i,j)∈P indicates two distinct bases $x_{i}x_{j}\in \{\text{CG},\;\;\text{GC},\;\;\text{AU},\text{UA},\text{GU},\text{UG}\}$ and each base only be paired at most once. A secondary structure is pseudoknot-free (i.e. nested pairs only) if there are no two pairs (i,j)∈P and (k,l)∈ P such that i<k<j<l. This work does not consider pseudoknots. P can also be represented as a dot-bracket string $y=y_{1}y_{2}\ldots y_{n}$, where a pair of indices (i,j)∈P corresponds to $y_{i}=“(”$ and $y_{j}=“)”$ and any unpaired index *k* corresponds to $y_{k}=“.”$. The unpaired indices in y are denoted as unpaired(y) and the set of paired indices in y is denoted as pairs(y), which equals P. For more details, see LinearFold (Huang *et al.* 2019) and LinearPartition (Zhang *et al.* 2020).

The *ensemble* of an RNA sequence x is the set of all secondary structures that x can possibly fold into, denoted as Y(x). The *free energy (change)* $\Delta G^{{}^{\circ}}(x,y)$ to characterizes the stability of y∈Y(x). The lower $\Delta G^{{}^{\circ}}(x,y)$, the more stable the structure y for x.

**Figure 1:**
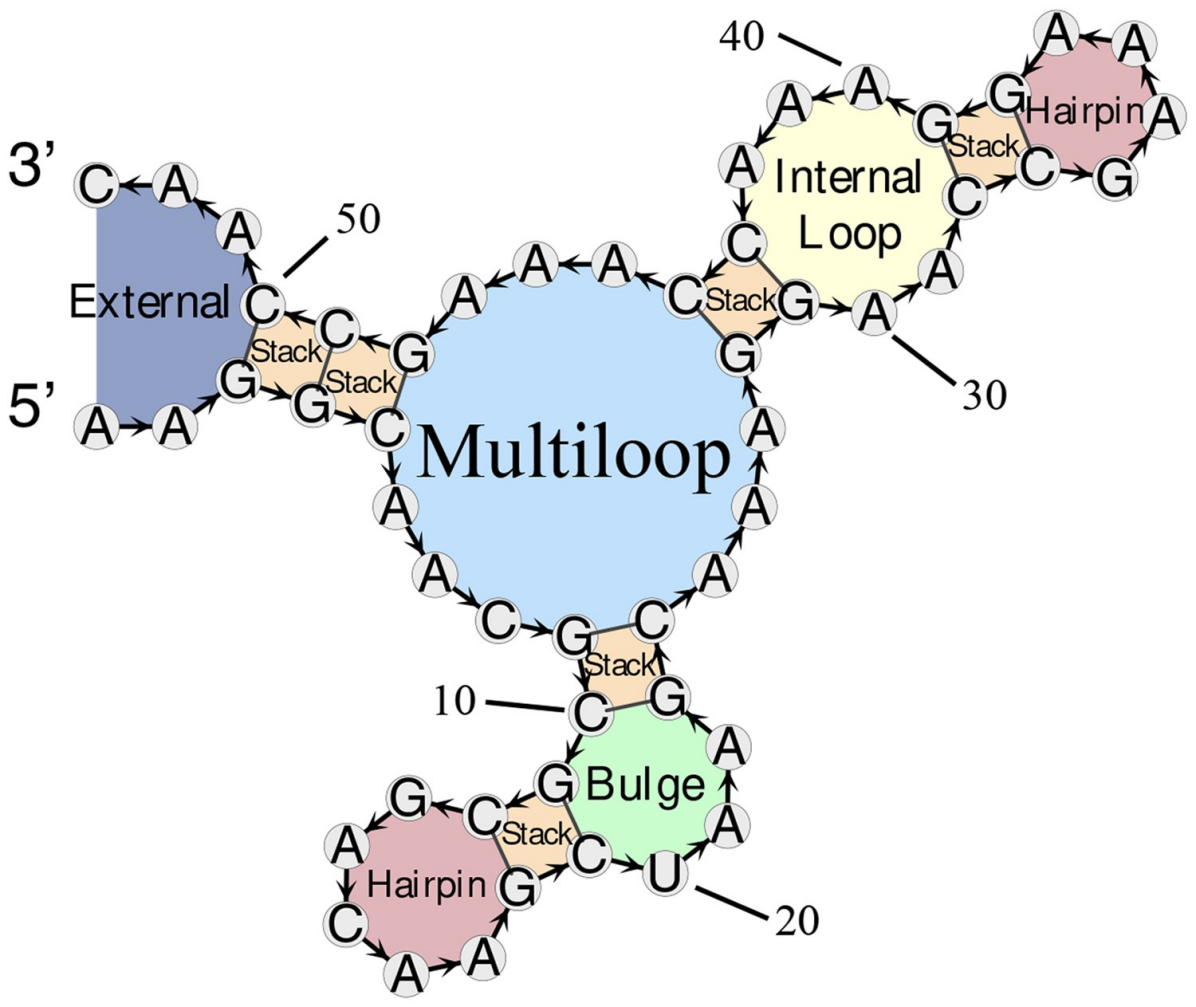
A secondary structure and its loops (including stacks).

A structure y is composed of a set of loops denoted as loops(y) where stacks of adjacent base pairs are also considered special loops, as illustrated in Fig. 1. The free energy change of a secondary structure y is the sum of the free energy change of each loop, i.e.


(1) $$\Delta G^{{}^{\circ}}(x,y)=\sum_{z\in \mathrm{loops}(y)}\Delta G^{{}^{\circ}}(x,z),$$

where each term $\Delta G^{{}^{\circ}}(x,z)$ is the energy for loop z. The energy of each loop is typically determined by nucleotides on the positions of enclosing pairs and their adjacent mismatch positions, which are named as *critical positions* and denoted as critical(z). See Section 1, available as supplementary data at *Bioinformatics* online for a detailed explanation of different loop types and their critical positions. The structure with the *minimum free energy* is the most stable structure in the ensemble Y(x). The minimum free energy of Y(x) is defined as


(2) $$\text{MFE}(x)=\underset{y\in Y(x)}{\min}\Delta G^{{}^{\circ}}(x,y).$$

The partition function sums up the Boltzmann terms of all structures,


(3) $$Q(x)=\sum_{y\in Y(x)}e^{-\Delta G^{{}^{\circ}}(x,y)/RT},$$

which defines the equilibrium probability or Boltzmann probability of a structure y in the ensemble


(4) $$p(y|x)=\frac{e^{-\Delta G^{{}^{\circ}}(x,y)/RT}}{Q(x)}=\frac{e^{-\Delta G^{{}^{\circ}}(x,y)/RT}}{\sum_{y^{\prime }\in Y(x)}e^{-\Delta G^{{}^{\circ}}(x,y^{\prime })/RT}}.$$

A (structural) *motif* is defined as a contiguous set of loops in a structure, which generalizes RNA structures [see Section 3, available as supplementary data at *Bioinformatics* online or FastMotif (Zhou *et al.* 2024) for details]. The thermodynamic definitions in Equations (1), (2), and (4) can be naturally extended to motifs. For example, the equilibrium probability of a motif m in the ensemble of a (partial) sequence x is


(5) $$p(m|x)=\frac{e^{-\Delta G^{{}^{\circ}}(x,m)/RT}}{Q(x)}=\frac{e^{-\Delta G^{{}^{\circ}}(x,m)/RT}}{\sum_{m^{\prime }\in M(x)}e^{-\Delta G^{{}^{\circ}}(x,m^{\prime })/RT}},$$

where M(x) is the motif ensemble of (partial) sequence x.

## 3 RNA designability: MFE versus Probabilistic

We first review the MFE-based designability before moving on to probabilistic designability.

### 3.1 MFE-based designabiity

Given a target structure $y^{⋆}$, MFE-based RNA design aims to find a suitable RNA sequence x such that $y^{⋆}$ is an MFE structure of x. We define X(y) to be the design space of structure y, which is the set of all RNA sequences that might fold into y, i.e. X(y)={x∣y∈Y(x)}. Structure $y^{⋆}$ is undesignable by the MFE criterion if and only if


(6) $$\;\forall x\in X(y),\exists y^{\prime }\neq y^{⋆},\Delta G^{{}^{\circ}}(x,y^{\prime })<\Delta G^{{}^{\circ}}(x,y),$$

which means for any sequence x there is always a rival structure $y^{\prime }$ that is energetically more favored than $y^{⋆}$.

Similarly, following the unique MFE (uMFE) criterion from previous studies (Haleš *et al.* 2015, Yao *et al.* 2019, Ward *et al.* 2023, Zhou *et al.* 2023), a structure $y^{⋆}$ is undesignable by the (uMFE) criterion if and only if


(7) $$\;\forall x\in X(y),\exists y^{\prime }\neq y^{⋆},\Delta G^{{}^{\circ}}(x,y^{\prime })\leq \Delta G^{{}^{\circ}}(x,y),$$

which means for any sequence x there is always a rival structure $y^{\prime }$ that is energetically no worse than $y^{⋆}$.

### 3.2 Probabilistic designability

MFE-based designability cannot quantify how likely or how easily a target structure can possibly form in the ensemble of any RNA sequence. To address this problem, we aim to find an upper bound pbound(y) such that


$$\mathrm{pbound}(y)\geq \underset{x}{\max}p(y|x).$$

While it is hard to obtain the exact value of $\max_{x}\;p(y|x)$, the tighter pbound(y) is, the better we know about the probabilistic designability of y. Similarly, we can also find an upper bound pbound(m) for a motif m such that $\mathrm{pbound}\geq (m)\max_{x}\;p(m|x)$.

## 4 Ensemble approximation via rival search

Rival structures (motifs) play a pivotal role in the previous works RIGEND (Zhou *et al.* 2024) and FastMotif (Zhou *et al.* 2025), which can prove RNA structures (motifs) undesignable under the uMFE criterion. The central idea is to identify a small set of rival structures (motifs)


$$Y_{r}=\{y_{1},y_{2},\ldots ,y_{k}\},y^{⋆}\notin Y_{r},$$

such that


(8) $$\forall x,\exists y^{\prime }\in Y_{r},\Delta G^{{}^{\circ}}(x,y^{\prime })\leq \Delta G^{{}^{\circ}}(x,y^{⋆}).$$

In other words, for every RNA sequence x, at least one explicitly identified rival structure is energetically no worse than the target structure. This condition guarantees that the target structure can never be the unique minimum free energy conformation of any sequence and is therefore uMFE-undesignable.

While effective, both RIGEND and FastMotif treat rival structures as *independent* competitors. Consequently, the collective thermodynamic effect of multiple rival structures is left unexploited. In this section, we introduce a new theory of Boltzmann ensemble approximation that leverages the collective contribution of rival structures to derive an upper bound on the equilibrium probability of the target structure.

To make the derivation intuitive, we begin with the simplest case, in which the ensemble is approximated by the target structure plus a single rival structure. We then extend this theory to the general case, where the ensemble approximation consists of multiple rival structures (motifs). Since motifs are generalizations of structures, we present the theory using structures for notational simplicity; all results apply to motifs directly.

### 4.1 Single rival structure (motif)

**Figure 2:**
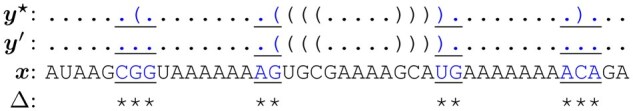
Ensemble approximation with a single rival structure.

We illustrate the idea using the Eterna100 structure “Simple Single Bond” (we cut off trailing unpaired bases to fit the page). When attempting to design this target structure $y^{⋆}$ (Fig. 2), the resulting sequence x consistently folds into a different but structurally very similar conformation $y^{\prime }$.

As proven in RIGEND (Zhou *et al.* 2024), it turns out that the free energy change difference between $y^{\prime }$ and $y^{⋆}$


$$\Delta \Delta G^{{}^{\circ}}(x,y^{\prime },y^{⋆})\overset{\Delta }{=}\Delta G^{{}^{\circ}}(x,y^{\prime })-\Delta G^{{}^{\circ}}(x,y^{⋆})$$

is only dependent on a number of *differential positions* $\Delta (y^{\prime },y^{⋆})$ (e.g. pairs and mismatches), defined as


$$\Delta (y^{\prime },y^{⋆})\overset{\Delta }{=}\underset{z\in \mathrm{loops}(y^{⋆})⊖l\mathrm{oops}(y^{\prime })}{\cup }\mathrm{critical}(z).$$

where ⊖ denotes the symmetric difference between two sets.

Figure 2 annotates the differential positions. Consequently, to verify that $\Delta \Delta G^{{}^{\circ}}(x,y^{\prime },y^{⋆})<0$ holds for all sequences x, it suffices to enumerate nucleotide assignments restricted to $\Delta (y^{\prime },y^{⋆})$ (The maximum size of $\Delta (y^{\prime },y^{⋆})$ or enumeration is constrained to bound complexity or running time.). This reduction enables an efficient and interpretable proof that $y^{⋆}$ is undesignable under the uMFE criterion. Beyond the uMFE undesignability, however, we notice that this process yields additional thermodynamic insight. In particular, rather than merely establishing the sign of $\Delta \Delta G^{{}^{\circ}}(x,y^{\prime },y^{⋆})$, we can compute the quantity,


$$\underset{x}{\max}\Delta \Delta G^{{}^{\circ}}(x,y^{\prime },y^{⋆}),$$

which represents the guaranteed energy advantage of the rival structure over the target across all sequences. This quantity directly leads to an upper bound on the equilibrium probability of the target structure:


(9) $$\begin{array}{c} p(y^{⋆}|x)=\frac{e^{-\Delta G^{{}^{\circ}}(x,y^{⋆})/RT}}{\sum_{y\in Y(x)}e^{-\Delta G^{{}^{\circ}}(x,y)/RT}}\\ \leq \frac{e^{-\Delta G^{{}^{\circ}}(x,y^{⋆})/RT}}{e^{-\Delta G^{{}^{\circ}}(x,y^{⋆})/RT}+e^{-\Delta G^{{}^{\circ}}(x,y^{\prime })/RT}}\\ =\frac{1}{1+e^{-(\Delta G^{{}^{\circ}}(x,y^{\prime })-\Delta G^{{}^{\circ}}(x,y^{⋆}))/RT}}. \end{array}$$

Taking the maximum over all sequences x, we obtain the following upper bound:


(10) $$\underset{x}{\max}p(y^{⋆}|x)\leq \frac{1}{1+e^{-\underset{x}{\max}\Delta \Delta G^{{}^{\circ}}(x,y^{\prime },y^{⋆})/RT}}.$$

Algorithm 1 shows how to identify an upper bound for $\max_{x}p(y^{⋆}|x)$ using a single rival structure $y^{\prime }$, which can be obtained by first taking a sequence x designed for $y^{⋆}$ and then folding x (assume MFE(x) is close to $y^{⋆}$). Note that ⊢ denotes the projection of a sequence x onto a set of positions ($\Delta (y^{\prime },y^{⋆})$). (see Section 2, available as supplementary data at *Bioinformatics* online)

Algorithm 1 Ensemble approximation with a single rival structure (motif).

**function** Ensemble Approximation($y^{⋆}$, $y^{\prime }$)

$$\Delta \Delta G_{\max}^{{}^{\circ}}\leftarrow \underset{\hat{x}\in \{x⊢\Delta (y^{\prime },y^{⋆})|x\in X(y^{⋆})\}}{\max}\Delta \Delta G^{{}^{\circ}}(\hat{x},y^{\prime },y^{⋆})$$

 **return**$\frac{1}{1+e^{-\Delta \Delta G_{\max}^{{}^{\circ}}/RT}}$ ▹ upper bound $\mathrm{pbound}(y^{⋆})$

### 4.2 Multiple rival structures (motifs)

Algorithm 1 is effective and interpretable, but the resulting bound is limited by the coarse approximation of the ensemble using only one rival structure. Incorporating more rival structures will yield a tighter approximation, though enumerating the full ensemble is infeasible.

**Figure 3:**
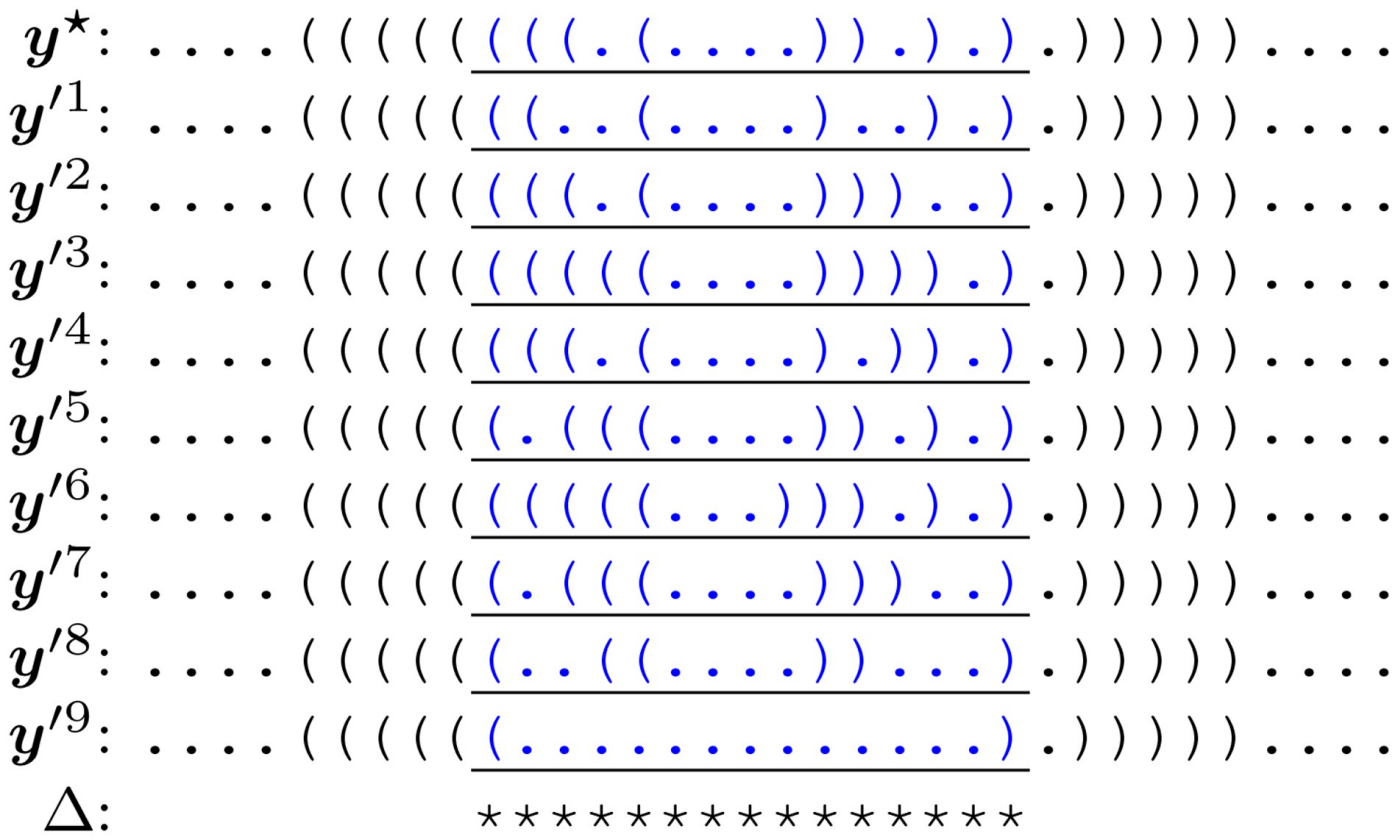
Ensemble approximation with nine rival structures.

We therefore generalize the framework to include multiple rival structures. Figure 3 illustrates this idea using the Eterna100 structure “Zigzag Semicircle.” Our previous work (Zhou *et al.* 2024) proved this structure uMFE-undesignable by identifying nine rival structures


$$\forall x,\exists y^{\prime }\in Y_{r}=\{y^{\prime 1},y^{\prime 2},\ldots ,y^{\prime 9}\},s.t.\;\Delta \Delta G^{{}^{\circ}}(x,y^{\prime },y^{⋆})\leq 0.$$

Rather than selecting a single rival, we include all nine rivals in the ensemble approximation to obtain a tighter bound. Specifically,


(11) $$\begin{array}{c} p(y^{⋆}|x)\leq \frac{e^{-\Delta \Delta G^{{}^{\circ}}(x,y^{⋆})/RT}}{e^{-\Delta \Delta G^{{}^{\circ}}(x,y^{⋆})/RT}+\sum_{y^{\prime }\in Y_{r}}e^{-\Delta \Delta G^{{}^{\circ}}(x,y^{\prime })/RT}}\\ \leq \frac{1}{1+\sum_{y^{\prime }\in Y_{r}}e^{-(\Delta \Delta G^{{}^{\circ}}(x,y^{\prime })-\Delta \Delta G^{{}^{\circ}}(x,y^{⋆}))/RT}}\\ \leq \frac{1}{1+\sum_{y^{\prime }\in Y_{r}}e^{-\Delta \Delta G^{{}^{\circ}}(x,y^{\prime },y^{⋆})/RT}}\\ \leq \frac{1}{1+e^{-\Delta \Delta G^{{}^{\circ}}(x,Y_{r},y^{⋆})/RT}}, \end{array}$$

where $e^{-\Delta \Delta G^{{}^{\circ}}(x,Y_{r},y^{⋆})/RT}\overset{\Delta }{=}\sum_{y^{\prime }\in Y_{r}}e^{-\Delta \Delta G^{{}^{\circ}}(x,y^{\prime },y^{⋆})/RT}$ summarizes the collective energetic dominance of the rival set over the target structure. Taking the maximum over all sequences yields


(12) $$\underset{x}{\max}p(y^{⋆}|x)\leq \frac{1}{1+e^{-\underset{x}{\max}\Delta \Delta G^{{}^{\circ}}(x,Y_{r},y^{⋆})/RT}}.$$

As in the single-rival case, exhaustive enumeration over all sequences is unnecessary. Owing to the sparsity of loop energy contributions in the Turner model, it suffices to enumerate nucleotides on the *overall differential positions*


(13) $$\Delta (Y_{r},y^{⋆})=\cup_{y^{\prime }\in Y_{r}}\Delta (y^{\prime },y^{⋆}).$$

Algorithm 2 outlines the resulting procedure to compute the upper bound using multiple rival structures.

Algorithm 2 Ensemble approximation with multiple rival structures (motifs).

**function** Ensemble Approximation($y^{⋆}$, $Y_{r}$)

$$\;\Delta \Delta G_{\max}^{{}^{\circ}}\leftarrow \underset{\hat{x}\in \{x⊢\Delta (Y_{r},y^{⋆})|x\in X(y^{⋆})\}}{\max}\Delta \Delta G^{{}^{\circ}}(\hat{x},Y_{r},y^{⋆})$$

 **return**$\frac{1}{1+e^{-\Delta \Delta G_{\max}^{{}^{\circ}}/RT}}$ ▹ upper bound $\mathrm{pbound}(y^{⋆})$

### 4.3 Complexity analysis

Algorithm 1 is a special case of Algorithm 2 with a single rival structure. The computational complexity of Algorithm 2 is determined by the size of the differential positions $\Delta (Y_{r},y^{⋆})$ defined in Equation (13), as the algorithm enumerates all nucleotide assignments restricted to these positions. Specifically, the time complexity is


(14) $$O\left(6^{\left|\mathrm{pairs}(\Delta (Y_{r},y^{⋆}))\right|}\cdot 4^{\left|\mathrm{unpaired}(\Delta (Y_{r},y^{⋆}))\right|}\right),$$

where paired positions admit six canonical base-pair types and unpaired positions admit four nucleotides.

In practice, to control runtime, we sample rival structures (motifs) by folding sequences compatible with the target structure (motif) and impose an upper limit on the number of differential assignments enumerated. If this limit is exceeded and no suitable rival structures can be sampled, the corresponding structure or motif is skipped, trading completeness for efficiency.

## 5 Linear-time dynamic programming over exponentially many decompositions

### 5.1 Structure and probability decomposition

**Figure 4:**
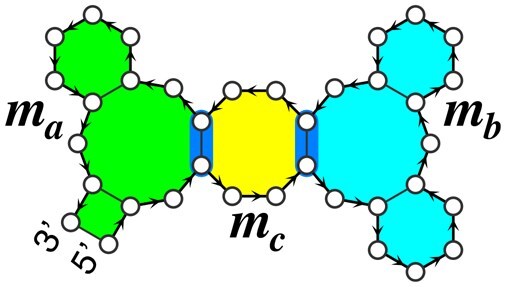
Example of structure decomposition for the Eterna100 structure “multilooping fun,” which is decomposed into 3 motifs $m_{a},m_{b},\text{and}\;m_{c}$ highlighted in different colors.

FastMotif (Zhou *et al.* 2025) shows that MFE-based structure undesignability can often be attributed to *local* (minimal) undesignable motifs. For example, Fig. 4 decomposes the Eterna100 structure *multilooping fun* into three motifs $m_{a}$, $m_{b}$, and $m_{c}$; in this instance, either $m_{a}$ or $m_{b}$ alone can certify the uMFE-undesignability of the full structure, providing a more localized and interpretable explanation than relying solely on global rival structures.

The principle that global designability is constrained by local designability also extends to *probabilistic* designability. CountingDesign (Yao 2021) proved that for any motif m contained in a target structure $y^{⋆}$, the motif probability upper-bounds the structure probability:


(15) $$\forall m\in y^{⋆},p(m|x)\geq p(y^{⋆}|x).$$

Applying this result to Fig. 4 yields, e.g. $p(y^{⋆}|x)\leq \;p(m_{a}|x)$ and $p(y^{⋆}|x)\leq p(m_{b}|x)$.

However, CountingDesign bounds $p(y^{⋆}|x)$ by the probability of a *single* motif. The joint impact of multiple non-overlapping motifs remains unexplored. Here we show that when a structure can be decomposed into non-overlapping motifs, the structure probability is upper-bounded by the *product* of the motif probabilities.

Theorem 1 (Probability decomposition over non-overlapping motifs).

*If a pseudoknot-free structure* $y^{⋆}$*can be decomposed into a set of non-overlapping motifs* $M=\{m_{1},m_{2},\ldots ,m_{C}\}$*, then for any sequence* $x\in X(y^{⋆})$,

(16) $$p(y^{⋆}|x)\leq \prod_{m\in M}p(m|x).$$

**Figure 5:**
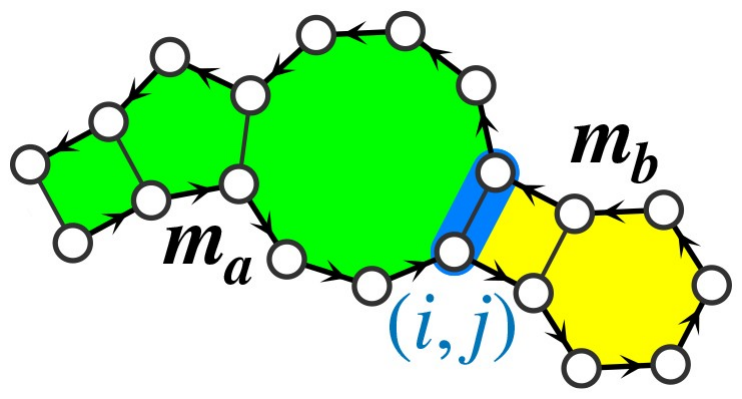
A motif m is split into $m_{a}$ and $m_{b}$ at the base pair (i,j).

***Proof.*** As a pseudoknot-free structure can be decomposed recursively, it suffices to prove the binary splitting step. Suppose a motif m is split into two submotifs $m_{a}$ and $m_{b}$ at a boundary base pair (i,j), as illustrated in Fig. 5. Let $P_{i,j}$ denote the event that bases *i* and *j* are paired. By the chain rule of probability,

(17) $$\begin{array}{c} \;\;p(m|x)=p(m_{a}+m_{b}|x)\\ =p(P_{i,j}|x)\times p(m_{a}|x,P_{i,j})\times p(m_{b}|x,P_{i,j})\\ =p(P_{i,j}|x)\times p(m_{a}|x)\times p(m_{b}|x)\\ \leq p(m_{a}|x)\times p(m_{b}|x). \end{array}$$

In a pseudoknot-free structure, the boundary pair (i,j) ensures that the two submotifs $m_{a}$ and $m_{b}$ are independent (no interactions between them) in the Boltzmann ensemble, which justifies the third line. The fourth line holds because the boundary pairs are enforced in the motif-level folding. ▪

Corollary 1
(18) $$\mathrm{pbound}(y^{⋆})\leq \prod_{m\in M}\mathrm{pbound}(m).$$

This corollary suggests that we can tighten the probability upper bound for a structure by exploiting probability bounds of smaller local motifs. For sufficiently small motifs, we can obtain an exact bound by enumerating all compatible (partial) sequences and folding them under the motif constraints. For motifs that are too large for exhaustive enumeration, we instead apply the motif ensemble approximation with rival motifs developed in the previous section.

**Figure 6:**
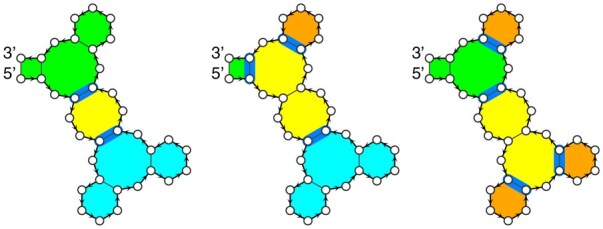
Three different decompositions for the same structure for the same structure shown in Fig. 4. Within each decomposition, different motifs are highlighted in different colors.

Figure 6 shows three alternative decompositions of the same Eterna100 structure from Fig. 4. In fact, a given structure admits exponentially many decompositions, because each base pair may or may not be selected as a splitting boundary. This yields the following Theorem.

Theorem 2. *The total number of distinct decompositions of a secondary structure* y*is* $2^{\left|\mathrm{pairs}(y)\right|}$.

Let M denote the set of decompositions that can be evaluated efficiently for a target structure $y^{⋆}$. The best probability bound obtainable via decomposition is then


(19) $$\mathrm{pbound}(y^{⋆})\leq \underset{M\in M}{\min}\prod_{m\in M}\mathrm{pbound}(m).$$

### 5.2 Optimal decomposition search by linear-time dynamic programming

Although enumerating all decompositions is intractable, exploring more decompositions can only tighten the resulting upper bound. The key question is therefore how to search a large space of decompositions efficiently.

**Figure 7:**
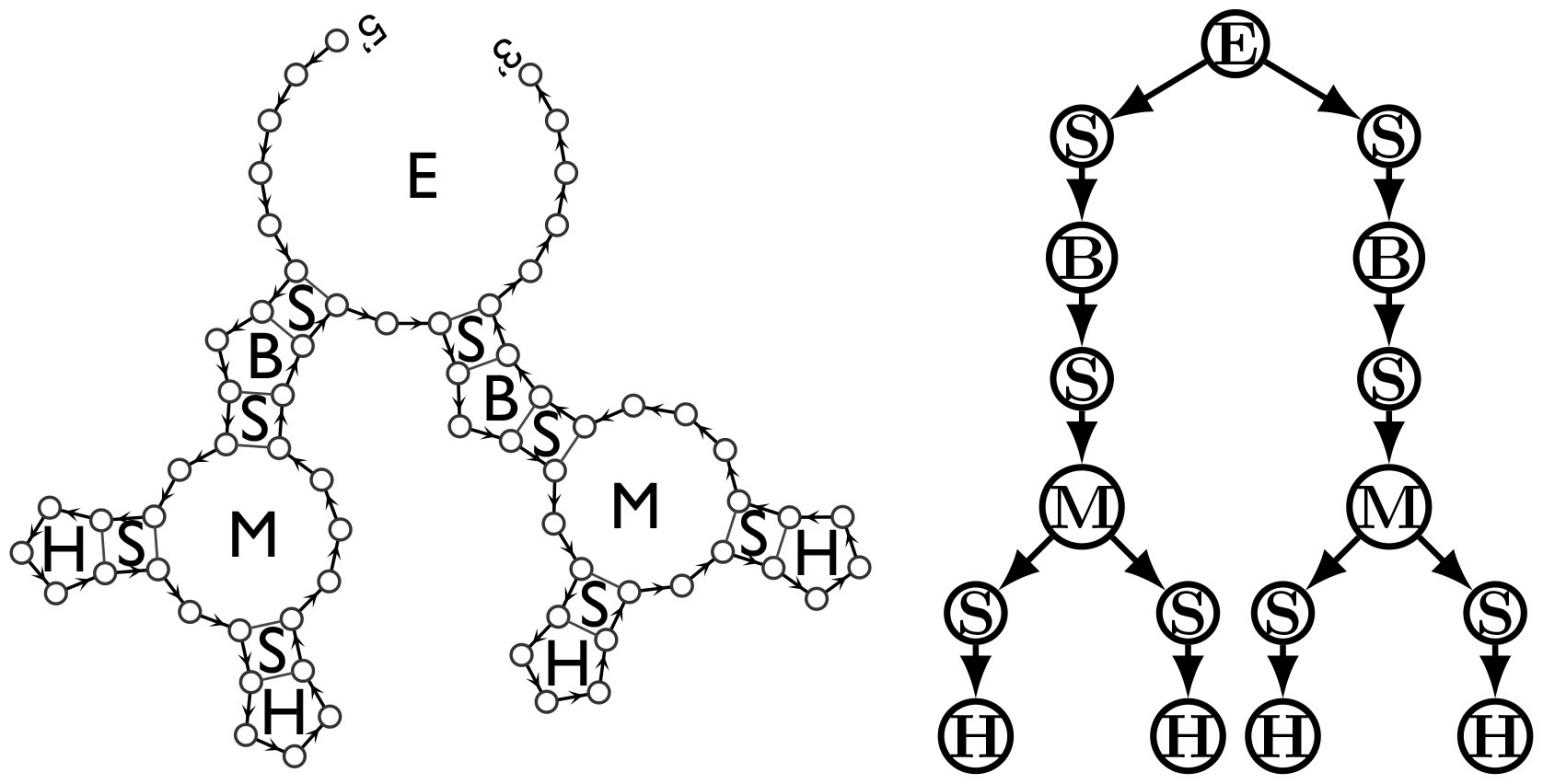
Left: the Eterna100 structure *Chicken Feet*. Right: corresponding loop tree where each loop (including stacking) is represented as a node. E, S, B, H, M represent different loop types.

**Figure 8:**
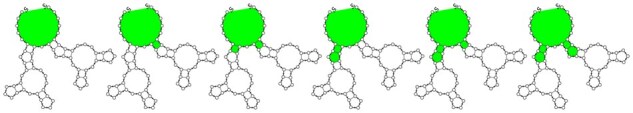
Examples of motif candidates generated at the root node. For demonstration, here the maximum values for *depth*, *width*, and *number of loops* are set as 3, 2, and 5, respectively, and 6 out of 9 cases are shown. In experiments the maximum *depth* is set as 5.

Inspired by dynamic programming techniques from compilers (Aho and Ullman 1972, Aho and Johnson 1976) and machine translation (Huang *et al.* 2006), we propose a linear-time memoized top-down algorithm (Algorithm 3) that explores many decompositions while remaining computationally efficient. We represent a target structure by a fixed loop tree τ, where each node corresponds to a loop in the structure (Fig. 7). We decompose the structure by traversing τ top-down, starting at the root. When visiting a loop node η, we are solving the subproblem for the subtree $\tau_{\eta }$, and we must choose a motif $m_{\eta }$ that contains η and some of its descendants. To avoid enumerating all possibilities, we constrain candidate motifs using three parameters: *depth*, *width*, and *number of loops*. Figure 8 shows examples of motif candidates generated when visiting the external loop at the root; the candidate-generation procedure is described in Algorithm 5 (in Section 4, available as supplementary data at *Bioinformatics* online). Once $m_{\eta }$ is selected, we recursively process each adjacent descendant loop node $\eta_{i}$ in the subtree $\tau_{\eta }$. This procedure yields the following dynamic programming recurrence:


(20) $$\mathrm{best}(\tau_{\eta })=\underset{m_{\eta }\in M\text{OTIF }G\text{EN}(\eta )}{\min}p\mathrm{bound}(m_{\eta })\prod_{\eta_{i}\in \mathrm{descendents}(m_{\eta })}\mathrm{best}(\tau_{\eta_{i}}),$$

where $\mathrm{best}(\tau_{\eta })$ represents the tightest upper bound for the substructure rooted at the loop node η. The base case occurs when η is a hairpin loop, for which $\mathrm{best}(\tau_{\eta })=1$.

Each loop node may admit many candidate motifs; the number of possible decompositions can also be calculated by dynamic programming recurrence:


(21) $$\mathrm{count}(\tau_{\eta })=\sum_{m_{\eta }\in \mathrm{MOTIF}\text{GEN}(\eta )}\prod_{\eta_{i}\in \mathrm{descendents}(m_{\eta })}\;\mathrm{count}(\tau_{\eta_{i}}),$$

with the base case value of 1 (leaf nodes). As a result, the decomposition possibilities grows exponentially with the size of the loop tree. We address this via memoization: each subtree rooted at a loop node is solved at most once and cached. We also store backpointers to recover the best motif choice at each node, and reconstruct the optimal decomposition by backtracking. The resulting dynamic program runs in O(αβ) time, where α is the number of loop nodes in the loop tree and β is the maximum number of motif candidates considered at any node.

Algorithm 3 Top-down memoized decomposition.

1: **function**Decompose(η)

2:  **if**cache[η] defined **then** 

3:    **return**cache[η]

4:  best←1

5:  candidates←MOTIF GEN(η,depth,width,max_loop)

6:  **for**$m_{\eta }\in \mathrm{candidates}$**do**▹ try each candidate $m_{\eta }$

7:    $\mathrm{product}\leftarrow \mathrm{pbound}(m_{\eta })$ ▹ exact or approx.

8:    descendants← loop nodes adjacent to m in $\tau_{\eta }$

9:    **for**$\eta_{i}\in \mathrm{descendants}$**do** 

10:      $\left(p_{i},m_{\eta_{i}}^{\text{best}})\leftarrow \text{DECOMPOSE}(\eta_{i}\right)$ ▹ recursion

11:      $\mathrm{product}\leftarrow \mathrm{product}\cdot p_{i}$

12:    **if**product<best**then** 

13:      best←product

14:      $m_{\eta }^{\text{best}}\leftarrow m_{\eta }$ ▹ plug in the results

15:  $\mathrm{cache}[\eta ]\leftarrow (\mathrm{best},m_{\eta }^{\text{best}})$ ▹ memoization

16:  **return**cache[η] ▹ optimal decomposition choice

## 6 Evaluation results

### 6.1 Implementation

Our work, *LinearDecompose*, consists of two core components: (i) Linear-Time Dynamic Decomposition (Algorithm 3) and (ii) Ensemble Approximation (Algorithm 2). The first module efficiently searches for the optimal motif decomposition by the recurrence formula Equation (20) over exponentially many candidates. The second module estimates probability bounds $\mathrm{pbound}(y^{⋆})$ or $\mathrm{pbound}(m^{⋆})$ for a target structure or motif.

Our C++ code runs on Linux machines with 4.0 GHz CPUs and 32 GB memory. It calls ViennaRNA (v2.7) folding energy engine (Lorenz *et al.* 2011) with Turner 2004 parameters (Mathews *et al.* 2004). Ensemble approximation is parallelized with OpenMP over 90 cores.

During decomposition search, Algorithm 3 repeatedly queries motif probability bounds $\mathrm{pbound}(m_{\eta })$ (line 8). To improve efficiency, we precompute and cache motif bounds using a hybrid strategy: (i) exact bounds via exhaustive enumeration for motifs of length up to 14, following CountingDesign (Yao 2021) and (ii) non-exact bounds via ensemble approximation for larger motifs.

### 6.2 Datasets and baselines

We evaluate on two popular RNA structure datasets:

1. **ArchiveII** (Cannone *et al.* 2002, Sloma and Mathews 2016) contains native RNA secondary structures spanning 10 families of naturally RNAs, including tRNA, rRNA, etc. We remove pseudoknotted structures and those incompatible with ViennaRNA’s default loop constraints. We only use structures longer than 200 *nt*, resulting in 1144 structures.
2. **Eterna100** (Anderson-Lee *et al.* 2016) consists of 100 secondary structures of varying design difficulty designed by human players of Eterna and is widely used for evaluating RNA design and designability.

We compare our method against CountingDesign and our enhanced version of it, CountingDesign+:

1. **CountingDesign** (Yao *et al.* 2019, Yao 2021) can identify exact probability bounds for very short motifs (up to 14 nucleotides) by exhaustively enumerating RNA sequences for each motif. However, the method is not scalable and lacks explainability.
2. **CountingDesign+**. We extend CountingDesign to include motifs with external loops, thus expanding their motif definition to align with ours.

### 6.3 Overall and individual results

Table 1 reports average probability bounds on both datasets. For reference, we also include the highest $p(y^{⋆}|x)$ achieved by the state-of-the-art RNA design methods, taking the better of SAMFEO (Zhou *et al.* 2023) and SamplingDesign (Tang *et al.* 2026) for each puzzle.

**Table 1:** Average probability bounds on each dataset (bold values represent the best metrics).

Dataset		ArchiveII	Eterna100
No. of unique structures		1144	100
Best achieved $p(y^{⋆}|x)↑$		0.766	0.599
$\mathrm{pbound}(y^{⋆})↓$	CountingDesign	0.896±0.242	0.898±0.234
	CountingDesign+	0.875±0.255	0.876±0.250
	LinearDecompose	**0.800**±0.288	**0.741**±0.318
	no ensemble approx.	0.866±0.256	0.839±0.275
	no dynamic decomp.	0.819±0.286	0.787±0.295

LinearDecompose consistently yields the tightest bounds, improving over CountingDesign+ by 0.075 on ArchiveII and 0.135 on Eterna100. This improvement is expected, as LinearDecompose is theoretically guaranteed to produce bounds no worse than CountingDesign+.

We also observe a clear correlation between probability bounds and empirical designability. ArchiveII structures are generally more designable: the average bound (0.800) is close to the achieved probability (0.766). In contrast, Eterna100 structures are less designable, with a larger gap between the achieved probability (0.599) and the bound (0.741). This difference is statistically significant (Mann–Whitney U = 72 687, *P* = 6.95 × 10^−6^, r=0.27).

**Figure 9:**
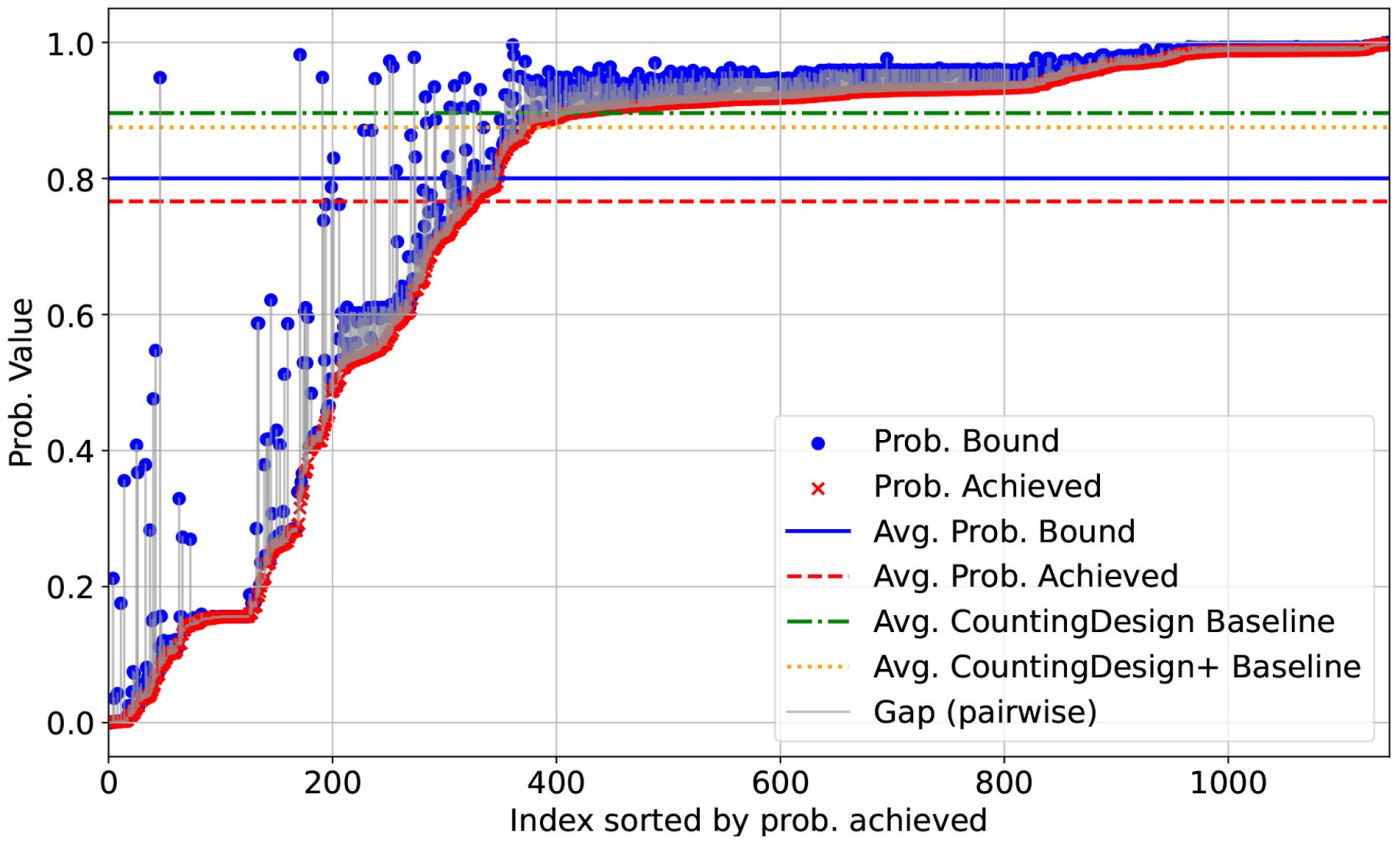
Probability bounds versus achieved $p(y^{⋆}|x)$ on ArchiveII.

**Figure 10:**
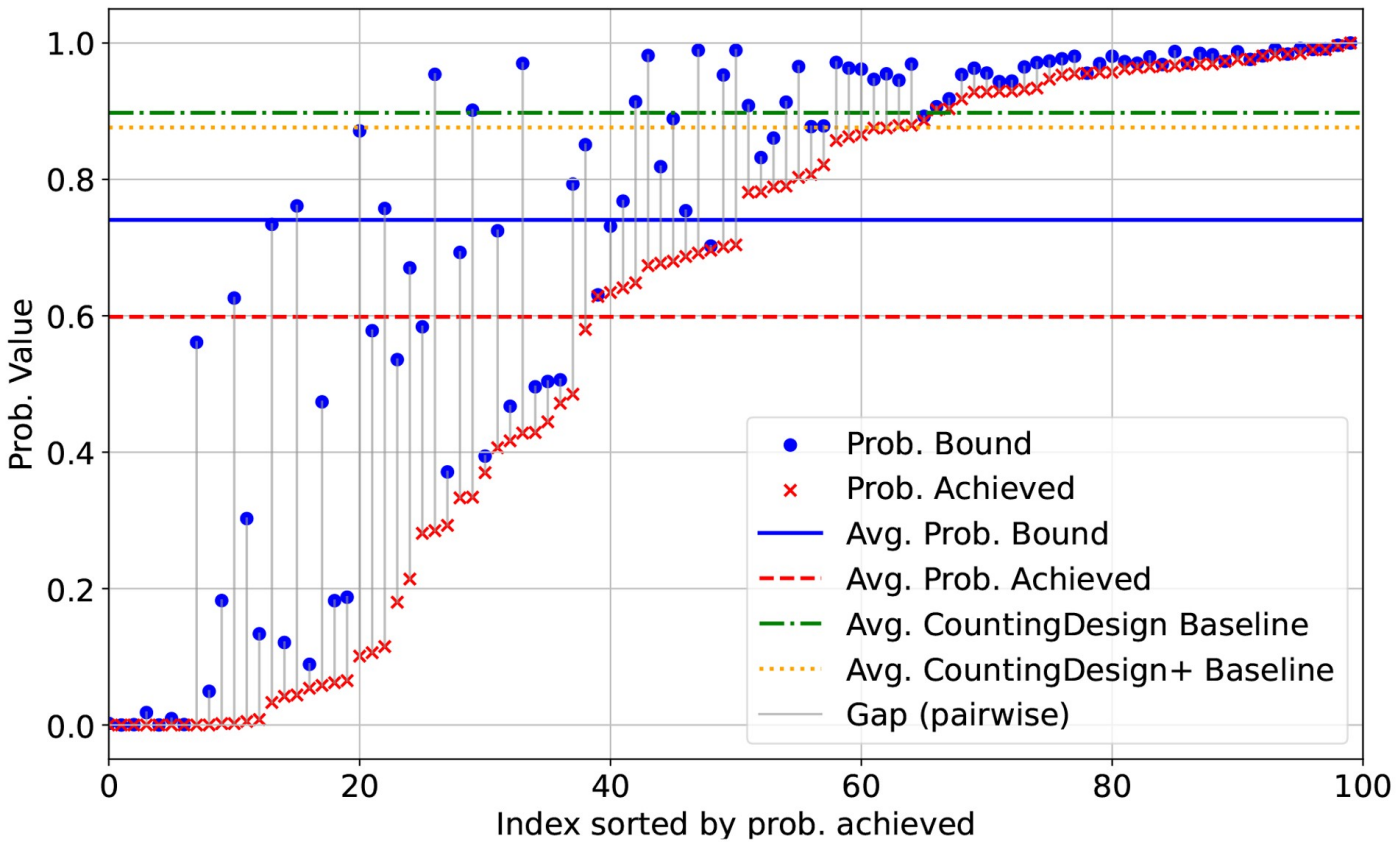
Probability bounds versus achieved $p(y^{⋆}|x)$ on Eterna100.

Figures 9 and 10 show per-structure bounds and best achieved $p(y^{⋆}|x)$. Bounds tend to be tighter for both highly designable and highly undesignable structures. Highly designable structures typically consist of motifs with uniformly high local probabilities, while highly undesignable structures often contain multiple motifs with very low bounds, often leading to near-zero global bounds.

These bounds also provide a useful lens for evaluating RNA design results. For example, a structure achieving probability 0.7 may be closer to its theoretical limit than another achieving 0.8 if its bound is substantially lower.

Table 2 presents a structural dissection of several Eterna100 puzzles previously shown to be uMFE-undesignable (Zhou *et al.* 2025). For each structure $y^{⋆}$, we report the number of decompositions explored by LinearDecompose [via dynamic programming Equation (21)], the total number of possible decompositions (via Theorem 2), the optimal decomposition achieving the tightest probability bound, the resulting global probability upper bound $\mathrm{pbound}(y^{⋆})$, and the best $p(y^{⋆}|x)$ achieved by SAMFEO and SamplingDesign (as a lowerbond).

**Table 2:** Structure dissection with Eterna100 puzzles.

Puzzle ($y^{⋆}$)	Decomposition		$\mathrm{pbound}(y^{⋆})$
	Explored	Optimal	max $p(y^{⋆}|x)$
Mat—elements and sections	47 012 668 out of 2^26^ = 67 108 864	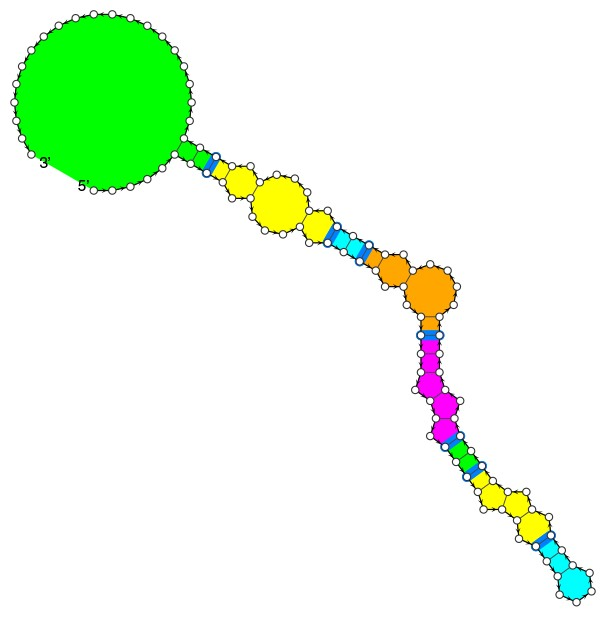	0.0889↓ 0.0426↑
Loop next to a multiloop	2 997 104 out of $2^{22}$= 4 194 304	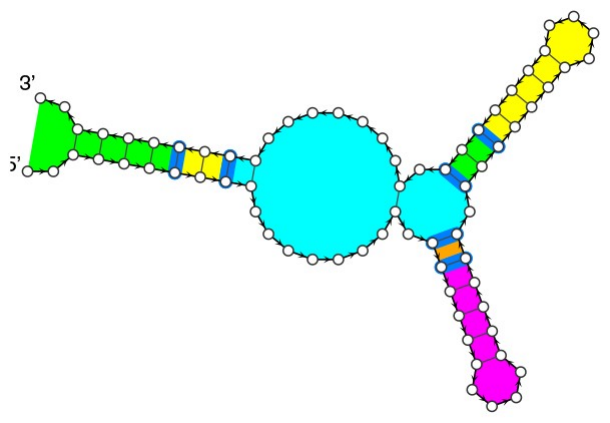	0.4958↓ 0.4293↑
Simple single bond	32 out of $2^{5}=32$	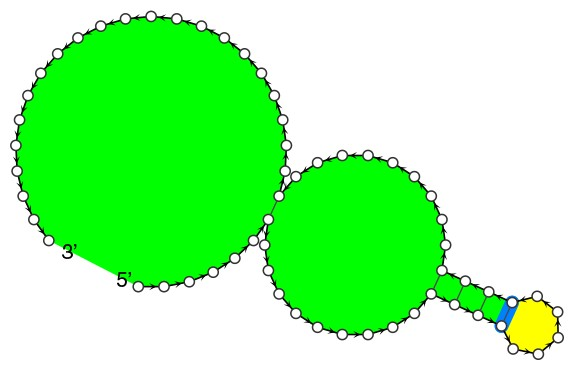	0.3942↓ 0.3700↑
1, 2, 3, and 4 bulges	2 734 333 out of $2^{22}$= 4 194 304	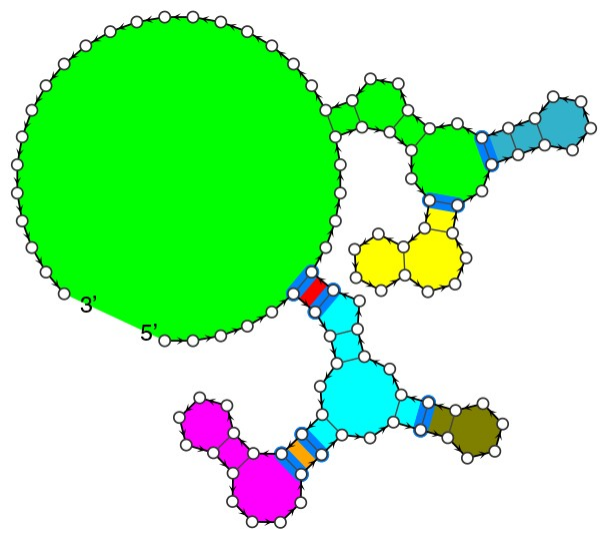	0.3026↓ 0.0054↑
Repetit. Seqs. 8/10	860 out of 2^10^ = 1024	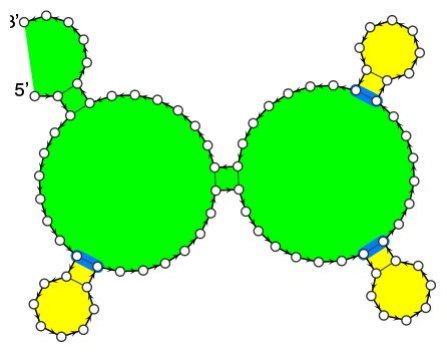	0.1874↓ 0.0651↑
Multilooping fun	119 out of $2^{7}=128$	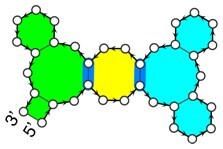	0.0006↓$2\times 10^{-6}$↑
Chicken feet	52 114 out of 2^16^ = 65 536	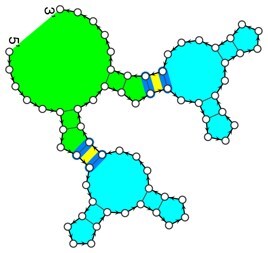	0.1336↓ 0.0080↑

LinearDecompose efficiently explores exponentially many decompositions per puzzle (e.g. 47M out of 67M total decompositions for the first puzzle), and consistently identifies a small set of local motifs whose combined bounds dominate the global probability bound. Notably, structures with complex multiloop or bulge configurations (e.g. *multilooping fun*) exhibit extremely low bounds, indicating that probabilistic undesignability can often be traced to a few highly constraining motifs. In addition, we also noticed that motifs with isolated base pairs tend to have low probability bounds, consistent with our prior systematic study (Zhou *et al.* 2026) on undesignable motifs that isolated base pairs are energetically unfavorable and often avoided in natural RNA structures. These results highlight the value of decomposition-based analysis for pinpointing the structural origins of low RNA designability.

### 6.4 Ablation studies

To assess the two core components of LinearDecompose, we perform two ablations: (i) disable motif ensemble approximation by setting approximate motif bounds to 1 and (ii) disable dynamic decomposition by using only the best single-motif bound. As shown in the last two rows of Table 1, both ablations lead to looser bounds, confirming the importance of both components. Nevertheless, the ablated variants still outperform the baselines in Table 1, demonstrating the robustness of the framework.

### 6.5 Efficiency

The runtime of LinearDecompose consists of decomposition search and motif ensemble approximation. Decomposition search is fast, averaging 0.01 s per structure on ArchiveII and 0.9 s on Eterna100. Ensemble approximation estimates bounds for 15 800 and 8000 unique motifs on ArchiveII and Eterna100, with an average cost of 7.6 and 10.7 s per motif, respectively. Approximately 10% and 2% of motifs on ArchiveII and Eterna100, respectively, are skipped due to exceeding the enumeration time limit, for which the bound defaults to 1. As these fractions are small, we expect the impact on overall bound quality to be minimal. The average time costs for ensemble approximation per structure are 106 s (ArchiveII) and 1039 s (Eterna100). The total runtimes are 106 and 1040 s per structure, respectively. Note that each motif bound can be cached once computed. As a result, the amortized runtime of LinearDecompose can be significantly reduced when analyzing multiple structures sharing common motifs, which is often the case in practice.

## 7 Discussion and conclusion

We introduced a theoretical framework based on ensemble approximation and probability decomposition to quantify the probabilistic designability of RNA secondary structures under any *factorizable* energy model or scoring function that decomposes onto individual loops. We further develop a linear-time dynamic-programming algorithm that efficiently searches for optimal decompositions among exponentially many choices. Together, the resulting bounds offer a novel, interpretable characterization of probabilistic designability: rather than a black-box score, each bound is explicitly supported by rival motifs that thermodynamically dominate the target, and the optimal decomposition identifies the specific motifs responsible for low global designability.

Applied to the ArchiveII and Eterna100 datasets, our algorithm LinearDecompose consistently produces tighter probability bounds under the Turner energy model than existing methods. Moreover, the resulting optimal decompositions offer interpretable explanations of structural design difficulty by identifying the specific motifs that limit global designability.

LinearDecompose still has some limitations:

1. Unless a structure is designed with a probability close to the probability bound found by LinearDecompose, the bound may be optimistic but loose.
2. Ensemble approximation is not universally applicable; for some structures or motifs, suitable rival motifs are hard to sample.

Future work includes improving rival motif generation to tighten ensemble approximations (see Appendix Section 5, available as supplementary data at *Bioinformatics* online for more details) and leveraging the optimal decomposition given by LinearDecompose as a guide for RNA design.

## Supplementary Material

btag246_Supplementary_Data

## Data Availability

Source code and data are available at https://github.com/shanry/RNA-Undesign.
