# Supplementary material for: Probabilistic RNA designability via interpretable ensemble approximation and dynamic decomposition
Source: Bioinformatics. 2026 Jul 7;42(Suppl 1):btag246. doi: 10.1093/bioinformatics/btag246 (PMC13340178; doi:10.1093/bioinformatics/btag246)
Supplement: btag246_Supplementary_Data [file btag246_supplementary_data.pdf]

Supplementary Information

S1. Structural Loops

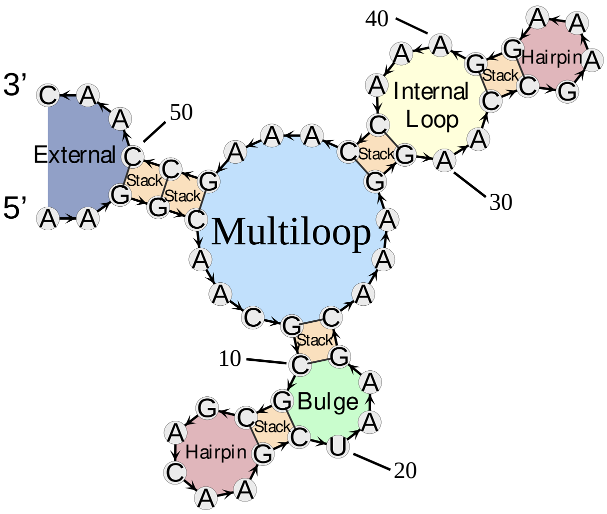

Fig. S1. An example of secondary structure and loops.

A secondary secondary structure can be decomposed into a collection of loops, where each loop is usually a region enclosed by base pair(s). Depending on the number of pairs on the boundary, main types of loops include hairpin loop, internal loop and multiloop, which are bounded by 1, 2 and 3 or more base pairs, respectively. In particular, the external loop is the most outside loop and is bounded by two ends ($5'$ and $3'$) and other base pair(s). Thus each loop can be identified by a set of pairs. Fig. S1 showcases an example of secondary structure with various types of loops, where the some of the loops are notated as

1. Hairpin: $H\langle(12, 18)\rangle$.
2. Bulge: $B\langle(10, 23), (11, 19)\rangle$.
3. Stack: $S\langle(3, 50), (4, 49)\rangle$.
4. Internal Loop: $I\langle(29, 43), (32, 39)\rangle$.
5. Multiloop: $M\langle(5, 48), (9, 24), (28, 44)\rangle$.
6. External Loop: $E\langle(3, 50)\rangle$.

Table S1. Critical positions of loops in Fig. 1

Loop Type	Critical Positions	
	Closing Pairs	Mismatches (Unpaired)
External	(3, 50)	2, 51
Stack	(3, 50), (4, 49)	
Stack	(4, 49), (5, 48)	
Multi	(5, 48), (9, 24), (28, 44)	4, 49, 8, 25, 27, 45
Stack	(9, 24), (10, 23)	
Bulge	(10, 23), (11, 19)	
Stack	(11, 19), (12, 18)	
Hairpin	(12, 18)	13, 17
Stack	(28, 44), (29, 43)	
Internal	(29, 43), (32, 39)	30, 42, 31, 40
Stack	(32, 39), (33, 38)	
Hairpin	(33, 38)	34, 37

The function $loops(\boldsymbol{y})$ is used to denote the set of loops in a structure $\boldsymbol{y}$. The free energy of a secondary structure $\boldsymbol{y}$ is the sum of the free energy of each loop,

$$\Delta G^{\circ}(\boldsymbol{x}, \boldsymbol{y}) = \sum_{\boldsymbol{z} \in loops(\boldsymbol{y})} \Delta G^{\circ}(\boldsymbol{x}, \boldsymbol{z}),$$

The energy of each loop is typically determined by nucleotides on the positions of enclosing pairs and their adjacent mismatch positions, which are named as *critical positions* in this article. Table S1 lists the critical positions for all the loops in Fig. 1 and Table S2 shows the indices of critical positions for each type of loops. Additionaly, some special hairpins of unstable triloops and stable tetraloops and hexaloops in Turner model have a separate energy lookup table. When evaluating the energy of a loop, it suffices to input only the nucleotides on its critical positions, i.e.,

$$\Delta G^{\circ}(\boldsymbol{x}, \boldsymbol{y}) = \sum_{\boldsymbol{z} \in loops(\boldsymbol{y})} \Delta G^{\circ}(\boldsymbol{x} \vdash critical(\boldsymbol{z}), \boldsymbol{z}), \qquad (22)$$

where $critical(\boldsymbol{z})$ denotes the critical positions of loop $\boldsymbol{z}$ and $\boldsymbol{x} \vdash critical(\boldsymbol{z})$ denotes the nucleotides from $\boldsymbol{x}$ that are "projected" onto $critical(\boldsymbol{z})$. The projection ($\vdash$) allows us to focus on the relevant nucleotides for energy evaluation. For instance,

$$critical(H\langle(12, 18)\rangle) = \{12, 13, 17, 18\}, \qquad (23)$$

$$critical(I\langle(29, 43), (32, 39)\rangle) = \{29, 30, 31, 32, 39, 40, 42, 43\}. \qquad (24)$$

For convenience, we also interchangeably put paired positions in brackets, i.e.,

$$critical(H\langle(12, 18)\rangle) = \{(12, 18), 13, 17\}, \qquad (25)$$

$$critical(I\langle(29, 43), (32, 39)\rangle) = \{(29, 43), (32, 39), 30, 31, 40, 42\}. \qquad (26)$$

Table S2. Critical positions for each type of loops under the Turner model implemented in ViennaRNA. Special hairpins (Mathews *et al.*, 2004) (triloops, tetraloops, and hexaloops) are not considered.

Loop Type	Critical Positions	
	Closing Pairs	Mismatches
External	$(i_1, j_1), (i_2, j_2), \ldots, (i_k, j_k)$	$(i_1 - 1, j_1 + 1), \ldots, (i_k - 1, j_k + 1)$
Hairpin	(i, j)	$i+1, j-1$
Stack	$(i, j), (k, l)$	–
Bulge	$(i, j), (k, l)$	–
Internal	$(i, j), (k, l)$	$i+1, j-1, k-1, l+1$
Multi	$(i, j), (i_1, j_1), \ldots, (i_k, j_k)$	$i+1, j-1, \ldots, i_k - 1, j_k + 1$

S2. Projection Operation

The projection operation is a key step in our ensemble approximation algorithm, which allows us to focus on the relevant nucleotides for energy evaluation. The projection of a sequence $\boldsymbol{x}$ onto a set of positions I is defined as follows:

Algorithm 4 Projection $\hat{\boldsymbol{x}} = \boldsymbol{x} \vdash I$

```
function PROJECTION(x, I)    ▷ I = [i_1, i_2, ..., i_n] is a list of critical positions
    x̂ ← map()                ▷ hash map
    for i in I do
        x̂[i] ← x_i           ▷ Map index i to nucleotide x_i
    return x̂
```

S3. Structural Motif

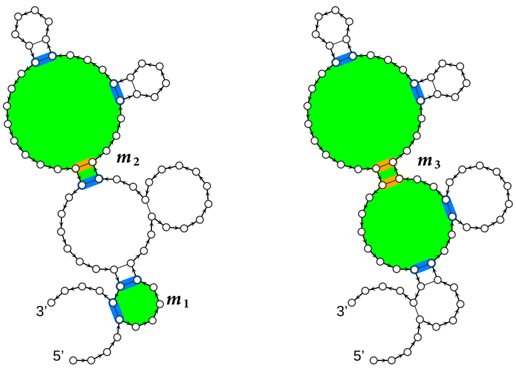

Fig. S2. Motifs of various cardinalities (numbers of loops): $card(\boldsymbol{m}_1) = 1$, $card(\boldsymbol{m}_2) = 2$, $card(\boldsymbol{m}_3) = 3$. Loops are highlighted in green, internal pairs (*ipairs*) in orange and boundary pairs (*bpairs*) in blue.

S3.1. Motif is a Generalization of Structure

Definition 1 A motif $\boldsymbol{m}$ is a contiguous (sub)set of loops in an RNA secondary structure $\boldsymbol{y}$, notated $\boldsymbol{m} \subseteq \boldsymbol{y}$.

Many functions defined for secondary structures can also be applied to motifs. For example, $loops(\boldsymbol{m})$ represents the set of loops within a motif $\boldsymbol{m}$, while $pairs(\boldsymbol{m})$ and $unpaired(\boldsymbol{m})$ represent the sets of base pairs and unpaired positions, respectively. We define the *cardinality* of $\boldsymbol{m}$ as the number of loops in $\boldsymbol{m}$, i.e., $card(\boldsymbol{m}) = |loops(\boldsymbol{m})|$. Fig. S2 illustrates three motifs, $\boldsymbol{m}_1, \boldsymbol{m}_2$, and $\boldsymbol{m}_3$, in a structure adapted from the Eterna puzzle "`Cat's Toy`". These motifs contain 1, 2, and 3 loops, respectively. We also define the *length* of a motif $|\boldsymbol{m}|$ as the number of bases it contains, which is consistent with the length of a secondary structure $|\boldsymbol{y}|$.

Since motifs are defined as sets of loops, we can conveniently use set relations to describe their interactions. A motif $\boldsymbol{m}_A$ is a *sub-motif* of another motif $\boldsymbol{m}_B$ if $\boldsymbol{m}_A$ is contained within $\boldsymbol{m}_B$, denoted as $\boldsymbol{m}_A \subseteq \boldsymbol{m}_B$. For the motifs in Fig. S2, we observe the relation $\boldsymbol{m}_2 \subseteq \boldsymbol{m}_3$. We further use $\boldsymbol{m}_A \subset \boldsymbol{m}_B$ to indicate that $\boldsymbol{m}_A$ is a proper sub-motif of $\boldsymbol{m}_B$, meaning $\boldsymbol{m}_A \neq \boldsymbol{m}_B$. Therefore, $\boldsymbol{m}_2 \subset \boldsymbol{m}_3$. The entire structure $\boldsymbol{y}$ can be regarded as the largest motif within itself, and accordingly, $\boldsymbol{m} \subseteq \boldsymbol{y}$ signifies that motif $\boldsymbol{m}$ is a part of structure $\boldsymbol{y}$, with $\boldsymbol{m} \subset \boldsymbol{y}$ implying $\boldsymbol{m}$ is strictly smaller than $\boldsymbol{y}$.

The loops in a motif $\boldsymbol{m}$ are connected by base pairs. Each base pair in $pairs(\boldsymbol{m})$ is classified as either an *internal pair* linking two loops in $\boldsymbol{m}$ or a *boundary pair* connecting one loop inside $\boldsymbol{m}$ to one outside. These two types of pairs in $\boldsymbol{m}$ are denoted as disjoint sets $ipairs(\boldsymbol{m})$ and $bpairs(\boldsymbol{m})$, respectively:

$$ipairs(\boldsymbol{m}) \cap bpairs(\boldsymbol{m}) = \varnothing, \;\; ipairs(\boldsymbol{m}) \cup bpairs(\boldsymbol{m}) = pairs(\boldsymbol{m}). \tag{27}$$

Utilizing the commonly accepted nearest neighbor model for RNA folding, it becomes evident that certain motifs may be absent from structures folded from RNA sequences. For instance, motif $\boldsymbol{m}_3$ in Fig. S2 is considered undesignable, as the removal of its two internal pairs consistently reduces the free energy. This brings us to the definition of an *undesignable motif*.

S3.2. Motif Ensemble from Constrained Folding

The designability of motifs is based on *constrained folding*. Given a sequence $\boldsymbol{x}$, a structure in its ensemble $\boldsymbol{y} \in \mathcal{Y}(\boldsymbol{x})$, we can conduct constrained folding by constraining the boundary pairs of $\boldsymbol{m}$, i.e., $bpairs(\boldsymbol{m})$. We generalize the concept of (structure) *ensemble* to *motif ensemble* as the set of motifs that $\boldsymbol{x}$ can possibly fold into (under the constraint $bpairs(\boldsymbol{m})$ being forced), denoted as $\mathcal{M}(\boldsymbol{x}, bpairs(\boldsymbol{m}))$. In the context of constrained folding,

the folding outcomes are unaffected by the nucleotides at the constrained positions. Thus, with slight notation abuse, we use $\boldsymbol{x}$ to denote a partial sequence corresponding to a motif $\boldsymbol{m}$, where each position in $\boldsymbol{x}$ matches a position in $\boldsymbol{m}$, and vice versa. By this definition, the motif ensemble of a partial sequence $\boldsymbol{x}$ is denoted as $\mathcal{M}(\boldsymbol{x})$. Similarly, the notation $\mathcal{X}(\boldsymbol{m})$ generalizes $\mathcal{X}(\boldsymbol{y})$ and represents all (partial) RNA sequences whose motif ensembles contain $\boldsymbol{m}$. Motifs in $\mathcal{M}(\boldsymbol{x}, bpairs\ (\boldsymbol{m}))$ have the same boundary pairs, i.e.,

$$\forall \boldsymbol{m}', \boldsymbol{m}'' \in \mathcal{M}(\boldsymbol{x}), bpairs(\boldsymbol{m}') = bpairs(\boldsymbol{m}'') = bpairs(\boldsymbol{m}). \tag{28}$$

The *free energy change* of a motif $\boldsymbol{m}$ is the sum of the free energy of the loops in $\boldsymbol{m}$,

$$\Delta G^\circ(\boldsymbol{x}, \boldsymbol{m}) = \sum_{\boldsymbol{z} \in loops(\boldsymbol{m})} \Delta G^\circ(\boldsymbol{x}, \boldsymbol{z}). \tag{29}$$

The definitions of MFE and uMFE can also be generalized to motifs via constrained folding.

Definition 2 A motif $\boldsymbol{m}^\star \subseteq \boldsymbol{y}$ is an MFE motif of folding $\boldsymbol{x}$ under constraint $bpairs(\boldsymbol{m})$, i.e., MFE$(\boldsymbol{x}, bpairs(\boldsymbol{m}))$, if and only if

$$\begin{aligned} &\forall \boldsymbol{m} \in \mathcal{M}(\boldsymbol{x}, bpairs(\boldsymbol{m})) \text{ and } \boldsymbol{m} \neq \boldsymbol{m}^\star, \\ &\Delta G^\circ(\boldsymbol{x}, \boldsymbol{m}^\star) \leq \Delta G^\circ(\boldsymbol{x}, \boldsymbol{m}). \end{aligned} \tag{30}$$

Definition 3 A motif $\boldsymbol{m}^\star \subseteq \boldsymbol{y}$ is an uMFE motif of folding $\boldsymbol{x}$ under constraint $bpairs(\boldsymbol{m})$, i.e., uMFE$(\boldsymbol{x}, bpairs(\boldsymbol{m}))$, if and only if

$$\begin{aligned} &\forall \boldsymbol{m} \in \mathcal{M}(\boldsymbol{x}, bpairs(\boldsymbol{m})) \text{ and } \boldsymbol{m} \neq \boldsymbol{m}^\star, \\ &\Delta G^\circ(\boldsymbol{x}, \boldsymbol{m}^\star) < \Delta G^\circ(\boldsymbol{x}, \boldsymbol{m}). \end{aligned} \tag{31}$$

Similarly, the equilibrium probability of a sequence folding into the motif is defined as,

$$\begin{aligned} p(\boldsymbol{m} \mid \boldsymbol{x}) &= \frac{e^{-\Delta G^\circ(\boldsymbol{x},\boldsymbol{m})/RT}}{Q(\boldsymbol{x})} \\ &= \frac{e^{-\Delta G^\circ(\boldsymbol{x},\boldsymbol{m})/RT}}{\sum_{\boldsymbol{m}' \in \mathcal{M}(\boldsymbol{x}, bpairs(\boldsymbol{m}))} e^{-\Delta G^\circ(\boldsymbol{x},\boldsymbol{m}')/RT}}. \end{aligned} \tag{32}$$

S4. Additional Pseudocode

The motif generation procedure used in LinearDecompose is shown in Algorithm 5, which generates candidate motifs for each loop node based on the specified constraints of depth, width, and number of loops.

S5. Discussion on the Tightness of Ensemble Approximation

The tightness of the probability bound produced by ensemble approximation (Algorithms 1 and 2) depends on two key factors: the *quality* and the *number* of rival structures (motifs).

Algorithm 5 Constrained Motif Generation from a Loop

```
function MotifGen(η, max_depth, max_width, max_loop)
    candidates ← ∅
    for d ← 1 to max_depth do
        M_d ← ∅                    ▷ motifs of depth d to be generated
        if d = 1 then
            M_d ← {η}              ▷ the motif is a single node η
        else
            for m ∈ M_{d−1} do
                m_new ← Grow(m)
                if Width(m_new) > max_width then
                    continue
                if |Loops(m_new)| > max_loop then
                    continue
                M_d ← M_d ∪ m_new
        candidates ← candidates ∪ M_d
    return candidates
```

S5.1. Quality of rival structures.

A rival structure is considered high quality if it thermodynamically dominates the target across a broad range of sequences, i.e., $\Delta\Delta G^\circ(\boldsymbol{x}, \boldsymbol{y}', \boldsymbol{y}^\star)$ is large and positive for many sequence assignments. Such rivals push the estimated bound down substantially. In contrast, a rival that only marginally outcompetes the target in a narrow region of sequence space contributes little to tightening the bound. In practice, rival quality is limited by the sampling strategy: rivals are obtained by folding sequences designed for the target, so they tend to be structurally similar to the target with small differential position sets $\Delta(\boldsymbol{y}', \boldsymbol{y}^\star)$. When the sampled rivals are structurally distant or energetically weak competitors, the resulting bound may be loose.

S5.2. Number of rival structures.

Including more rivals in the ensemble approximation directly tightens the bound, because each additional rival contributes an extra term to the denominator of the probability bound (Eq. 8). Intuitively, more competitors in the approximated ensemble means the target structure claims a smaller share of the Boltzmann weight. The bound can only decrease as rivals are added, so the method is conservative by construction. However, adding more rivals also increases the size of the overall differential positions $\Delta(Y_r, \boldsymbol{y}^\star)$ (Eq. 13), which exponentially increases the enumeration cost. In practice, we impose an upper limit on the enumeration size, which may prevent the bound from fully benefiting from a large rival set.

S5.3. When bounds may be loose.

Bounds tend to be loose when: (1) the sampled rivals are of low quality, e.g., they only weakly dominate the target; (2) the number of sampled rivals is small; or (3) the enumeration limit is reached before the best sequence assignment is found. These conditions are more likely for large structures with complex loop configurations, where the differential position set grows quickly and sampling diverse, high-quality rivals is harder.

S5.4. Utility for evaluating RNA design results.

Beyond certifying undesignability, the probability bounds provide a practical tool for evaluating and comparing RNA design results. Table 2 illustrates varying degrees of bound tightness across different Eterna100 puzzles. For example, *Simple Single Bond* achieves a best design probability of 0.370 against a bound of 0.394 — a tight gap indicating the structure is close to its theoretical limit and the design is near-optimal. In contrast, *1, 2, 3 and 4 bulges* achieves only 0.005 against a bound of 0.303, suggesting substantial room for improvement. Similarly, *multilooping fun* has a bound of 0.0006, consistent with the negligible best design probability of 2×10^{-6}, confirming that this structure is highly constrained and essentially undesignable at the ensemble level. These examples demonstrate that probability bounds provide a richer, quantitative characterization of design difficulty than binary undesignability criteria alone.

S6. Detailed Plots

The following figures show the probability bounds vs. achieved probabilities for all 1144 ArchiveII structures and 100 Eterna100 structures. In addition, the per-structure bounds from baselines (CountingDesign and CountingDesign+) are also scattered for comparison.

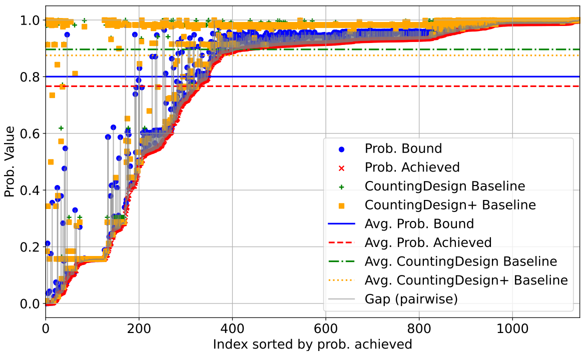

Fig. S3. Probability bounds vs. achieved $p(\boldsymbol{y}^\star \mid \boldsymbol{x})$ on ArchiveII.

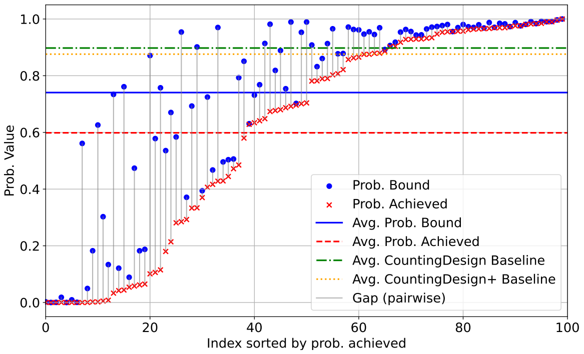

Fig. S4. Probability bounds vs. achieved $p(\boldsymbol{y}^\star \mid \boldsymbol{x})$ on Eterna100.
